# Diagnostic value of whole-body-focused ultrasonography in high-acuity patients in the emergency department: a prospective single-center cross-sectional study

**DOI:** 10.1186/s13089-019-0126-7

**Published:** 2019-05-27

**Authors:** M. Riishede, C. B. Laursen, L. S. Teglbjærg, E. Rancinger, P. B. Pedersen, S. M. Luef, J. H. Clausen, O. Graumann, A. T. Lassen, G. Baatrup

**Affiliations:** 1 The Research Section, Department of Surgery (A), Odense University Hospital, Baagoes Allé 15, 5700 Svendborg, Denmark; 2 Department of Clinical Research, University of Southern Denmark, SDU, Odense, Denmark; 3 Department of Internal Medicine & Emergency Medicine (M/FAM), Odense University Hospital, Svendborg, Denmark; 4 Department of Respiratory Medicine, Odense University Hospital, Odense, Denmark; 5 Department of Cardiology, Hospital of Southern Jutland, Aabenraa, Denmark; 6 Department of Radiology, Odense University Hospital, Odense, Denmark; 7 Department of Emergency Medicine, Odense University Hospital, Odense, Denmark

## Abstract

**Background:**

A fast and diagnostic accurate tool to assess the unselected category of high-acuity patients, in whom the underlying pathology is not always obvious, is needed in the emergency departments (ED). We aim to describe the feasibility, validity and diagnostic yield of a routine whole-body-focused ultrasonography (wbf-us) in an unselected group of high-acuity ED patients.

**Methods:**

In a prospective observational study, a convenience sample of ED patients (≥ 18 years) with a high-acuity score or systolic blood pressure < 100 mmHg received a routine wbf-us of the heart, lungs, abdomen and deep veins by two non-expert sonographers. Final diagnosis was established by experienced auditors. Investigators were blinded to the patients’ medical history and emergency physicians and auditors were blinded to the investigators assessments. Diagnostic accuracy was assessed by comparing the investigators’ ultrasonography findings to a structured double-blinded clinical audit of patient files.

**Results:**

We included 171 patients, initiated a whole-body-focused ultrasonography examination (wbf-us) in 160 and completed it in 128 patients with an average time of a full examination of 28 min. We found pathology in 65/171 (38%) of the patients whose most frequent symptoms upon arrival were cardiopulmonary. Among the patients who received wbf-us, we found the majority of pathology by wbf-us of the lungs (*n* = 50, 31%), the heart (*n* = 26, 16%), few in the abdomen (*n* = 5, 3%) and none in the deep veins. The overall sensitivity was 50–100%, specificity 84–94%, positive predictive value 11–44% and negative predictive value 94–100%.

**Conclusion:**

Focused cardiopulmonary ultrasonography might be considered for routine use in high-acuity ED patients with cardiopulmonary symptoms whereas focused ultrasonography of the abdomen and deep veins performed by non-expert sonographers only seems indicated in selected patients.

*Trial registration* Danish Data Protection Agency (ID 13/12076). Committee on Biomedical Research Ethics for the Region of Southern Denmark (ID S-20130047).

**Electronic supplementary material:**

The online version of this article (10.1186/s13089-019-0126-7) contains supplementary material, which is available to authorized users.

## Introduction

Rapid and accurate identification of the cause for severe illness in unselected high-acuity patients is often a challenge to the emergency physician (EP) [1, 2].

Focused ultrasonography (f-us) is a noninvasive bedside tool, generally free of pain and ionizing radiation, fairly easy to learn and associated with a reduction in the use of more cumbersome imaging [3–5]. The use of f-us in high-acuity patients has increased over the last decade [6].

Various studies have addressed symptoms using a range of single-organ f-us to whole-body f-us (wbf-us) in selected patients and shown that f-us can be both fast and accurate in identification of selected pathologies [7–9].

Moreover, studies have shown that f-us in selected patients can increase the diagnostic accuracy by increasing the sensitivity to up to 97% when adding f-us alongside standard clinical examination compared to a sensitivity as low as 50% by standard clinical examination alone indicating that f-us holds the potential to reduce the time to correct diagnosis and correct initial treatment [10].

Alongside these studies, evidence-based guidelines that describe indications, technical performance and interpretation of f-us as well as recommendations and guidelines of the implementation of f-us in ED or intensive care units have been published [11–13].

However, to our knowledge, the use of a wbf-us in an unselected group of high-acuity patients remains uninvestigated. Therefore, the aim of our study was to assess the feasibility (measured by duration and amount of evaluable wbf-us) and the validity and diagnostic yield (measured by sensitivity, specificity, negative and positive predictive values) of a wbf-us in an unselected group of high-acuity ED patients to clarify if the method of examination could be a helpful tool for the physician in the daily clinic. The wbf-us was performed by two physicians who are non-expert sonographers reflecting the daily clinical setting in most EDs.

## Methods

A descriptive prospective, cross-sectional study was performed in the ED at Odense University Hospital, Svendborg, Denmark, which has approximately 29,000 visits per year of which around 4100 are triaged as high-acuity patients upon arrival.

Acute admissions in Denmark are to public hospitals via emergency call or referred to by the general practitioner. Selected patients bypass the ED and are transported directly to an expert department (e.g. with signs of acute myocardial infarction, pregnancy or trauma to head, thorax or column).

Initial diagnostic tests ordered for patients admitted to the ED are in general based on symptoms and signs and include blood sample analyses, ECG, microbiological analyses and imaging (e.g. X-ray, CT, MR). Ultrasonography examinations are mainly performed by summoned specialists and are not an integrated part of the initial examination of ED patients [14].

The inclusion took place from December 2013 to March 2016. Included patients constituted a convenience sample; consequently, inclusion only took place immediately after the patients’ arrival to the ED. The investigators were blinded to the patients’ medical history and only aware of the patients’ direct cause of admittance upon inclusion. Patients were included after informed consent by the investigators if they were: (1) acutely admitted to the ED, (2) ≥ 18 years, (3) upon primary assessment they presented with a high-acuity score or systolic blood pressure < 100 mmHg. A high-acuity score was defined as the two highest levels of acuity in our ED triage system based on the patients’ initial clinical scores (e.g. saturation, respiratory rate, heart rate) (Additional file 1: Appendix S1). Exclusion criteria were patients with mental disability or patients unable to sign informed consent.

The included patients constituted a mixture of both surgical and medical etiology with symptoms and signs of various diseases (e.g. respiratory problems, cerebral thrombosis or hip fracture). The investigators performed the wbf-us blinded to the EP and only in his absence. The wbf-us was performed with a Philips CX 50 ultrasound machine (Philips, USA) and consisted of wbf-us of the lungs (Flus), the heart (Fcu), the abdomen (Faus) and the deep veins of the legs (Lcu). The wbf-us was always performed in the same sequence commenced by Flus then Fcu, Faus and Lcu and only aborted or paused on demand of the patient (e.g. due to pain or nausea) or in case of urgent treatment or supplemental analysis (e.g. scans, blood samples). The wbf-us was not completed if not resumed within 1 h and the investigation time was defined as the time spent on the actual ultrasound examination. The time used at wbf-us examination time was measured from the first to the last gaze with the ultrasonography probe. Interruptions were not included.

Wbf-us diagnoses were assessed according to predefined diagnostic criteria (Additional file 2: Appendix S2) and performed according to the following predefined f-us protocols.

Focused lung ultrasonography (Flus): performed with a L12-5 linear array transducer (Philips) (12–5 MHz) or a C5-1 curved array transducer (Philips) (5–1 MHz) using a modification of the principles described by Lichtenstein, Volpicelli and colleagues as described by Laursen et al. [15–17]. We looked for pneumothorax, interstitial syndrome and pleural effusion.

Focused cardiac ultrasonography (Fcu): performed with a S5-1 sector transducer (Philips) (5–1 MHz) using the focus assessed transthoracic echocardiography (Fate) protocol [18]. We looked for reduced ejection fraction (EF) and pericardial effusion.

Focused abdominal ultrasonography (Faus): performed with a C5-1 broadband curved array transducer (Philips) (5–1 MHz) using the three abdominal windows from the RUSH protocol and including a transverse and oblique window of the abdominal aorta [7]. We looked for free abdominal fluid and abdominal aorta aneurism.

Limited compression ultrasonography (Lcu): performed with a L12-5 linear array transducer (Philips) (12–5 MHz) or C5-1 curved array transducer (Philips) (5–1 MHz) according to the American College of Emergency Medicine’s imaging criteria compendium [19]. We looked for signs of a deep-vein thrombosis (DVT).

The investigators (ER and MR) had varying experience in wbf-us. Prior to inclusion MR performed: focused lung ultrasonography (Flus) > 80, Focused assessment with transthoracic echocardiography (Fate) > 50, focused assessment with sonography in trauma (Faus) > 40, limited compression ultrasonography (Lcu) > 50. Of these, 25 of each scanning area were supervised and evaluated during a period of f-us certification according to the national guidelines [13]. ER had no prior f-us skills and received an e-learn course of the wbf-us protocol including an oral presentation and hands on training followed by 5–10 supervised examinations until she was capable of performing the wbf-us protocol with an image quality sufficient for diagnostic evaluation (Additional file 2: Appendix S2).

A video clip from each window of the wbf-us examination (e.g. one respiratory cycle from each Flus window, 6 s of the heart from each Fcu projection) was saved to an external hard disc for later expert evaluations. With the exception of wbf-us findings of acute life-threatening conditions all wbf-us results were kept blinded from patients and EPs.

All wbf-us clips were finally assessed by expert physicians with European Federation of Societies for Ultrasound in Medicine and Biology (EFSUMB) for ultrasonography level 2–3 from specialties of respiratory medicine (Flus), cardiology (Fcu) and radiology (Faus, Lcu). The expert sonographers evaluated the investigators’ wbf-us examinations and assessed the final diagnoses blinded to the investigators’ interpretations and the EPs clinical evaluations.

All results from the wbf-us were compared to gold standard, which was defined as the final diagnoses assessed by blinded clinical audit of the medical record.

The blinded audit was performed according to predefined diagnostic criteria by two auditors who, independently of each other and blinded to all wbf-us clips and the interpretations of these, set the final diagnoses. In case of any discrepancy, a third auditor made the final decision (Additional file 3: Appendix S3).

### Analyses

Data were reported as proportions with a 95% confidence interval (CI) based at binominal distribution. Diagnostic performances were established using sensitivity, specificity, positive and negative predictive values (PPV, NPV), their 95% CI and Fleiss kappa coefficients (*K*) using the results from the blinded medical record audit as gold standard.

Descriptive statistics, describing medical history, medication and vital parameters upon admission were performed. Data analysis was conducted using Stata version 14.0 (Stata Corporation LP^®^, Texas, USA).

The main analyses were performed by the ‘intention to treat’ principle. Missing data in the basic characteristics were handled by simple imputation: we kept patients with no or incomplete wbf-us in the study. Any cases of image quality inadequate for evaluation were handled as follows: (a) handled as non-pathologic in the analysis of total wbf-us diagnoses, (b) left out in the analysis of diagnostic accuracy and inter-observer agreement.

### Ethics

Patients were included after informed consent. Any wbf-us that revealed signs of a predefined acute life-threatening condition was immediately non-blinded to the EP in charge (e.g. pericardial effusion, ejection fraction ≤ 45%, pulmonary edema, massive pleural effusion, pneumothorax, pulmonary emboli or deep-vein thrombosis (DVT). Subsequently, it was up to the clinician to assess whether further action should be taken. The study was undertaken in accordance with the Helsinki Declaration and approved by the Committee on Biomedical Research Ethics for the Region of Southern Denmark (ID S-20130047) and the Danish Data Protection Agency (ID 13/12076).

## Results

**Fig. 1:**
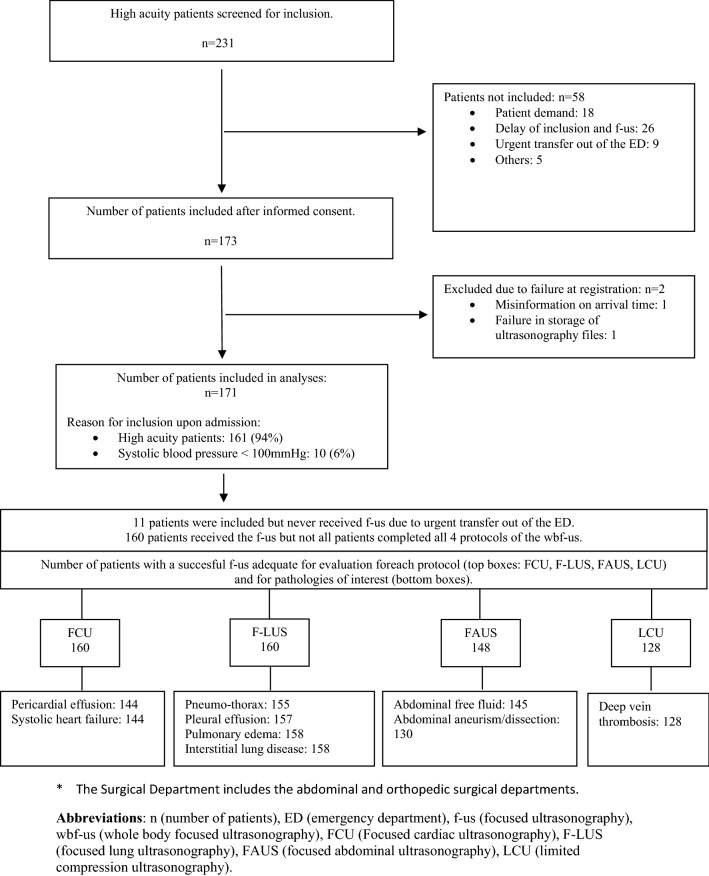
Flow chart of inclusion of adult high acuity patients admitted to the Emergency Department

We included 171 patients (Fig. 1). Median age was 69 years (range 18–97) and 94 (55%) were women (Table 1).

**Table 1:** Baseline characteristics of adult high acuity patients included upon admission to the emergency department

Characteristics (*n* = 171)	Value
Age (years), median (range)	69 (18–97)
Gender (*n* (%))	
Female	94 (55)
Male	77 (45)
Vital parameters upon admission	
Heart rate (beats/min) (*n* = 170), median (IQR)	87 (72–100)
Systolic BP (mmHg) (*n* = 170), median (IQR)	135 (119–153)
Diastolic BP (mmHg) (*n* = 170), median (IQR)	75 (65–87)
Respiration rate (breaths/min) (*n* = 164), median (IQR)	18 (16–21)
Saturation (%)	
Patients with oxygen supply (*n* = 34), median (IQR)	95 (93–98)
Saturation (%)	
Patients without oxygen supply (*n* = 152), median (IQR)	97 (93–99)
Temperature (°C) (*n* = 170), median (IQR)	37.0 (36.0–37.7)
Glascow Coma Score (*n* = 170), median (IQR)	15 (15–15)
Blood glucose (*n* = 144), median (IQR)	6.7 (6–8.6)
Former medical history among the 171 included patients, *n* (%)	
Coronary artery disease	11 (6)
Heart failure	29 (17)
Arterial hypertension	44 (25)
Diabetes mellitus	24 (14)
Chronic obstructive pulmonary disease	33 (19)
Asthma	15 (9)
Thromboembolic disease	14 (8)
Other Pulmonary disease	8 (5)
Psychiatric disorder	30 (18)
Stroke	6 (4)
Chronic kidney disease	7 (4)
Reason for inclusion upon admission among the 171 included patients, *n* (%)	
High acuity patients	161 (94)
Systolic blood pressure < 100 mmHg	10 (6)
Affiliated department upon admission among the 171 included patients, *n* (%)	
Medical	147 (86)
Surgical^a^	17 (10)
Others	7 (4)

Number of patients with an ultrasonography examination sufficient for diagnostic evaluation for each protocol (*N*) and for the individual pathology (*n*). Cases of no ultrasonography clips are left out

*Fcu* focused cardiac ultrasonography, *Flus* focused lung ultrasonography, *Faus* focused abdominal ultrasonography, *Lcu* limited compression ultrasonography

^a^Surgical includes the general and orthopedic surgical departments

With the wbf-us we found one or more pathologies in 65/171 (38%) patients allocated to the departments as follows: Medical Department 58/147 (39%); Surgical Departments (abdominal and orthopedic) 4/17 (24%) and from other departments 3/7 (43%).

Based on the wbf-us, we found most pathology by the protocols of Flus 50/160 patients (31%), Fcu 26/160 patients (16%) and few cases by Faus 5/148 patients (3%) and none by Lcu 0/128 patients (0%) (Table 2).

**Table 2:** Distribution of pathology found by whole body focused ultrasonography in adult high acuity ED patients

Diagnoses	Investigators’ prevalence of pathology in each subgroup, *n* (%)
Lungs (Flus): *N* = 160	
Pathological findings in the 50 patients with positive findings	
Pneumothorax, (of 155 investigated)	0 (0%)
Pleural effusion, (of 157 investigated)	39 (25%)
Pulmonary edema, (of 158 investigated)	25 (16%)
Interstitial lung disease, (of 158 investigated)	25 (16%)
Cardiac (Fcu): *N* = 160	
Pathological findings in the 26 patients with positive findings	
Pericardial effusion, (of 144 investigated)	10 (7%)
Systolic heart failure (EF < 45%), *n* = 144	19 (13%)
Abdomen (Faus): *N* = 148	
Pathological findings in the 5 patients with positive findings	
Free fluid abdomen, (of 145 investigated)	4 (3%)
Abdominal aorta aneurism/dissection^a^, (of 130 investigated)	1 (1%)
Veins (Lcu): *N* = 128	
Pathological findings in 0 patients	
Deep vein thrombosis, (of 128 investigated)	0 (0%)

*ED* Emergency Department

^a^Signs of aorta aneurism or dissection are defined as a deviation from the normal diameter (women < 2.9 cm, men < 3.2 cm) of the abdominal aorta

The most frequent specific pathological findings were pleural effusion *n* = 39, interstitial syndrome/pulmonary edema *n* = 25 and systolic heart failure *n* = 19. For prevalence of pathological wbf-us findings, see Table 2.

A wbf-us adequate to answer questions from all 4 scanning protocols could be performed in 128 (75%) patients with a median examination time of 28 min (IQR 24–32). The wbf-us was never initiated in 11 (6%) patients and not completed in 43 (25%) primarily due to acute clinical procedures (e.g. imaging and blood samples).

The investigators’ ultrasonography findings were confirmed by expert evaluations with an internal total agreement of 89–100%.

Final diagnostic gold standard was established by chart review by two independent researchers and had an overall inter-observer agreement of 97.6% (Kappa 0.74).

Based on the gold standard evaluation of the 171 included patients, we found pleural effusion in 22 (13%), interstitial syndrome 3 (2%), pulmonary edema in 6 (4%) and systolic heart failure in 4 (2%). For the complete list of final diagnoses, see Table 3 and Additional file 4.

**Table 3:** Gold standard defined as final diagnoses established by blinded audit of the medical records

Diagnoses (found in all 171 patients)	Number of final diagnosis, *n* (%)
Cerebral diseases	
Cerebral stroke	12 (7)
Cerebral hemorrhagia	2 (1)
Pulmonary diseases	
COPD	17 (11)
Suspected COPD with exacerbation	5 (3)
Asthma	4 (2)
Interstitial lung disease^a^	3 (2)
Pneumonia	24 (14)
Pulmonary oedema^a^	6 (4)
Pleural effusion^a^	22 (13)
Parapneumonic effusion	6 (4)
Empyema	0 (0)
Pulmonary embolism	3 (2)
Pneumothorax^a^	0 (0)
Heart diseases	
Systolic heart failure^a^	4 (2)
Nonsystolic heart failure	2 (1)
Myocardial infarction (acute or recent evolved)	4 (2)
Pericardial effusion^a^	2 (1)
Valvular heart disease	12 (7)
Infective endocarditis	0 (0)
Cardiac arrythmia	26 (15)
Chest pain (myocardial infarction ruled out)	9 (5)
Abdominal diseases	
Ileus	0 (0)
Appendicitis	2 (1)
Free abdominal fluid^a^	1 (1)
Pancreatitis/kidney stone	8 (5)
Dissection/aneurism of the abdominal aorta^a^	0 (0)
Orthopedic diseases	
Fracture	8 (5)
Luxation	5 (3)
Orthopedic injuries	6 (4)
Other diseases or symptoms	
DVT^a^	0 (0)
Anemia	14 (8)
Malignancy	7 (4)
Poisoning	4 (2)
Lipotymi/dizziness/discomfort	10 (6)
Infection with extrapulmonary focus	31 (18)
No diagnostic criteria met	28 (16)

Final diagnoses are established on all included patients, *n* = 171

Ultrasound examination clips deemed not evaluable by novice sonographers or experts are treated as normal in this analysis

*COPD* chronic obstructive pulmonary disease, *DVT* deep vein thrombosis

^a^Diagnoses for which we looked for in the whole body focused ultrasonography examination

Finally, we performed a ‘per protocol’ contingency table analysis of the correlation between the pathology found by wbf-us and gold standard diagnoses and found that according to the most frequent diagnostic findings the wbf-us revealed 100% of patients with interstitial lung disease (3/3) and pericardial effusion (2/2), 89% of patients with pleural effusion (17/19) and 50% of the patients with pulmonary edema (3/6) and systolic heart failure (2/4) (Table 4 and Additional file 5a, b).

**Table 4:** Per protocol: correlation and diagnostic accuracy between investigators pathological ultrasonography findings and gold standard pathology

Interstitial lung disease			
Investigator	Gold standard		Total
	1	0	
1	3	22	25
0	0	133	133
Total	3	155	158

Pericardial effusion			
Investigator	Gold standard		Total
	1	0	
1	2	8	10
0	0	134	134
Total	2	142	144

Pleural effusion			
Investigator	Gold standard		Total
	1	0	
1	17	22	39
0	2	116	118
Total	19	138	157

Pulmonary edema			
Investigator	Gold standard		Total
	1	0	
1	3	22	25
0	3	130	133
Total	6	152	158

Systolic heart failure			
Investigator	Gold standard		Total
	1	0	
1	2	17	19
0	2	123	125
Total	4	140	144

*PPV* positive predictive value, *NPV* negative predictive value, *CI* 95% confidence interval

This resulted in an overall sensitivity of 50–100%, specificity of 84–94%, PPV of 11–44% and a NPV of 94–100%. Diagnostic accuracy values and their 95% CI are provided in Table 5.

**Table 5:** Diagnostic accuracy of investigators pathology found with focused ultrasonography compared to gold standard

Diagnoses	Gold standard positive/f-us positive	Sensitivity % (95% CI)	Specificity % (95% CI)	PPV % (95% CI)	NPV % (95% CI)
Heart (Fcu): *N* = 160					
Pericardial effusion, *n* = 144	2/10	100 (16–100)	94 (89–8)	26 (3–56)	100 (97–100)
Systolic heart failure (EF < 45%), *n* = 144	4/19	50 (7–93)	88 (81–93)	11 (1–33)	98 (94–100)
Lungs (Flus): *N* = 160					
Pneumothorax^a^, *n* = 155	0/0				
Pleural effusion, *n* = 157	19/39	89 (67–99)	84 (77–90)	44 (28–60)	98 (94–100)
Pulmonary edema, *n* = 158	6/25	50 (12–88)	86 (79–91)	12 (3–31)	98 (94–100)
Interstitial lung disease, *n* = 158	3/25	100 (29–100)	86 (79–91)	12 (3–31)	100 (97–100)
Abdomen (Faus): *N* = 148					
Abdominal aorta aneurism/dissection^b^, *n* = 130	0/1				
Free fluid abdomen^c^, *n* = 145	1/4	100 (CI 3–100)	98 (CI 94–100)	25 (CI 1–81)	100 (CI 97–100)
Veins (Lcu): *N* = 128					
Deep-vein thrombosis, *n* = 128^a^	0/0				

Gold standard: final diagnoses of blinded medical record audit

Statistical analyses are made using the statistical concept ‘per protocol’: cases of no ultrasonography clips are left out

Number of patients with an ultrasonography examination sufficient for diagnostic evaluation for each protocol (*N*) and for each pathology (*n*)

*F-us* fo**c**used ultrasonography, *Fcu* focused cardiac ultrasonography, *Flus* focused lung ultrasonography, *Faus* focused abdominal ultrasonography, *Lcu* limited compression ultrasonography

^a^Due to a low number of events the sensitivity and specificity could not be calculated

^b^Signs of aorta aneurism or dissection are defined as a deviation from the normal diameter (women < 2.9 cm, men < 3.2 cm) of the abdominal aorta

^c^Free fluid abdomen’ is assessed from the 3 views of the abdominal f-us. If any region of the abdominal f-us showed any sign of free fluid the FAUS was regarded as pathological

## Discussion

We initiated wbf-us in 160 high-acuity ED patients in an unselected group of 171 patients and found pathology in a little more than 1/3 patients. The most frequent pathological ultrasonography findings were of cardiopulmonary origin and were mainly found in patients admitted with respiratory or circulatory symptoms.

Similar to other studies of f-us of the heart and lungs in dyspneic ED patients, we found cardiopulmonary diagnoses as heart failure, pneumonia and pleural effusion among the most common pathologies [10, 20, 21]. In parallel, our findings are consistent with the low number of free abdominal fluid, abdominal aorta aneurisms/dissection and DVT found by gold standard as well as the uneven distribution of patients to the medical (147) and surgical (17) departments.

The f-us examination time included f-us examination, filing of f-us clips and patient handling in relation to the f-us examination, and interruptions from other persons or procedures were not included. Comparable to similar studies of selected f-us of the heart, lungs ± the abdomen and the deep veins, we had an average time for a complete wbf-us of 28 min (IQR 28–32) [9, 20, 22, 23]. This made the examination susceptible to numerous interruptions due to the urgency of treatment.

Our study hereby shows that a routine wbf-us approach might not be pertinent in all high-acuity ED patients. It supports the idea of a more rational approach where Flus and Fcu are routinely applied in patients with signs or symptoms of disease in the chest and where Faus and Lcu might be relevant in selected patients.

### Diagnostic accuracy of the f-us examinations

The investigators found several pathologies that were not found by gold standard. The most common diagnoses were: (a) pericardial effusion in 8 patients, with a majority of final diagnoses of infection with extrapulmonary focus and pneumonia; (b) systolic heart failure in 17 patients with the majority of final diagnoses of cardiopulmonary origin; and (c) pleural effusion, pulmonary edema and interstitial lung disease in 22 patients with the majority of final diagnoses of cardiopulmonary origin.

The inter-observer agreement on 89–100% on the wbf-us images emphasizes that the investigators’ images are of acceptable quality.

Several studies of cardiopulmonary f-us has demonstrated that f-us is superior to the often used gold standard (chest radiography) when it comes to the detection of certain pathologies of acute cardiopulmonary disease [20, 24]. This increases the amount of false-positive f-us findings and leads to a drop in PPV which hereby becomes falsely low. A study of ED patients with respiratory symptoms demonstrated that f-us of the heart, lungs and deep veins in the legs increased the prescription of advanced diagnostic tests earlier in the hospital stay, which confirmed the suspected f-us diagnoses and identified missed life-threatening diseases. When compared to the control group during the entire stay at hospital, there was no significant difference in amount of prescribed advanced diagnostic tests [17]. Similar to these studies we find many false-positive results as we also find an overall diagnostic accuracy comparable with similar studies (Table 4) [8, 10, 17, 25, 26].

The investigators overlooked the pathology according to final diagnosis as follows: (a) systolic heart failure in two patients (one was due to strict diagnostic criteria, the other might be due to lack of skills among the investigators since the expert sonographer diagnosed the systolic heart failure when reviewing the f-us clips); (b) pleural effusion in two patients (expert evaluation found no pathology in one patient and deemed the f-us clips unevaluable in the other patient); and (c) pulmonary edema in three patients where the expert sonographer found no pulmonary edema either.

Similar to the previously mentioned studies we find that the wbf-us is a reliable tool to find pathology in highly acute ED patients as well as the wbf-us can find pathology that would otherwise be missed at clinical assessment [10, 17]. The f-us thereby holds the potential to reduce the time to correct diagnosis, proper treatment and the amount of X-rays we expose our patients to.

## Strengths and limitations

Recommendations and guidelines have been made to increase f-us competencies and uniformity of assessment [13, 27], however, several studies of individual f-us procedures show that even with a short introduction novices can assess patients with a diagnostic accuracy similar to that of experts [4, 25]. The wbf-us was performed in an unselected group of high-acuity patients in a clinically realistic ED setting which provided us the possibility to investigate if certain patient categories are redundant or missed in the search of who to perform the wbf-us on and which f-us modalities to use. To reduce the subjective bias often seen in blinded auditing, the final diagnoses were performed by three auditors. The third auditor only set a diagnosis in case of disagreement between the two first auditors whose agreement was 97.6% (Kappa 0.74).

The study also has limitations: being a single-center study performed by only two investigators, this study might not be generalizable to all other EDs. The basic characteristics in our study population mimic those found in similar studies in high-acuity ED patients [27]. However, the distribution of patients between the departments was uneven which mimics the patient category in tertiary hospital EDs, but it mainly gives us a picture of the wbf-us in medical ED patients.

Selection bias is present; the inclusion only took place when the investigators were present (daytime), some eligible patients were included but received no f-us due to urgent need of treatment in expert departments, others bypassed the ED due to administrative decisions and finally some patients were too ill to cooperate to f-us or to give informed consent. We believe that the selection bias could not be avoided.

The wbf-us protocol was designed to look for the most critical but reversible conditions that can be visualized by ultrasonography and that are most often found in high-acuity ED patients. The wbf-us protocol was also designed to be simple and fast so that it could be used by ED physicians who not all are expert sonographers. We, therefore, excluded a lot of pathologies from the wbf-us protocol that could have been relevant in some patients such as patients with abdominal pain (e.g. cholecystitis, ectopic pregnancy, pneumoperitoneum).

Despite these actions, the wbf-us examination was time consuming. In addition, the research context of the study increased the amount of data registration which increased the amount of interruptions due to other clinical procedures which again increased the number of non-completed f-us examinations (Fig. 1). Consequently, the feasibility of the wbf-us in the clinical setting is influenced by the duration of the scanning procedure.

Furthermore, the wbf-us diagnoses were based on predefined criteria but the investigators were not blinded to the patients’ clinical presentation and could have been influenced by the patients’ clinical appearance to increase or decrease the probability of distinctive pathological patterns.

However, final diagnoses could only be obtained if diagnosed or confirmed during the actual stay at hospital. Therefore, the high number of false-positive findings and the related low PPV is partly explained by the fact that patients admitted with other pathological conditions than those diagnosed at previous admissions (e.g. heart failure) had these well-known pathologies confirmed by the wbf-us. But if patients were asymptomatic and the EP did not take action on the unblinded f-us findings or found them relevant enough to be listed in the medical record, then these pathologies were not reconfirmed during the actual stay and, therefore, not included as a final diagnosis. Furthermore, some final diagnoses could have occurred during the hospital stay (e.g. pleural effusion or pulmonary edema) and not have been present at the patients arrival the ED where the wbf-us was performed. This could partly explain the unexpected poor diagnostic accuracy (low sensitivity and PPV) in these findings. Unfortunately, our study was not designed to take these factors into account.

Moreover, we performed a routine wbf-us in all included patients regardless their symptoms and found unexpected pathology (e.g. pleural effusion in a patient with hip fracture). The dilemma is that we do not know the clinical relevance of these unexpected ultrasonography findings in asymptomatic patients and our study is not designed to investigate this.

## Conclusion

F-us in the hands of non-expert sonographers seems to be a useful diagnostic bedside tool in adult high-acuity medical ED patients. However, Flus and Fcu seem indicated in patients with cardiopulmonary symptoms whereas Faus and Lcu seem indicated in selected patients. F-us holds the potential to reduce the time to correct diagnosis and to find pathology that is missed at initial diagnostic assessment.

## Additional files

**Additional file 1: Appendix S1.** Definition of high acuity patients included in this study.

**Additional file 2: Appendix S2.** Sonographic definitions and diagnostic criteria of the focused ultrasonography examination.

**Additional file 3: Appendix S3.** Blinded audit and audit diagnostic criteria of final diagnoses.

**Additional file 4.** Number of pathological ultrasonography findings compared to gold standard diagnoses in the adult high acuity patients admitted to the emergency department.

**Additional file 5.** **a** Per protocol correlation and diagnostic accuracy between investigators pathological ultrasonography findings and gold standard pathology found in adult high acuity patients admitted to the emergency department. Contingency tables and calculations are made according to the statistical concept ‘per protocol’, hence only including the patients who received a focused ultrasonography examination. **b** Per intention to treat correlation and diagnostic accuracy between investigators pathological ultrasonography and gold standard pathology found in adult high acuity patients admitted to the emergency department. Contingency tables and calculations are made according to the statistical concept `intention to treat´ including the entire study population. N = 171.

## Data Availability

A full study protocol can be assessed from the corresponding author. The datasets analyzed during the current study are available from the corresponding author on reasonable request.

## Abbreviations

CI: confidence interval
COPD: chronic obstructive pulmonary disease
DVT: deep-vein thrombosis
ED: Emergency Department
EP: Emergency Physician
Faus: focused abdominal ultrasonography
Fcu: focused cardiac ultrasonography
Flus: focused lung ultrasonography
IQR: inter quartile range
K: kappa coefficients
Lcu: limited compression ultrasonography
N: number of patients
NPV: negative predictive value
PPV: positive predictive value
