# Supplementary material for: Diagnostic value of whole-body-focused ultrasonography in high-acuity patients in the emergency department: a prospective single-center cross-sectional study
Source: Ultrasound J. 2019 May 27;11:11. doi: 10.1186/s13089-019-0126-7 (PMC6638611; doi:10.1186/s13089-019-0126-7)
Supplement: Supplementary file 1 — Additional file 1: Appendix S1. Definition of high acuity patients included in this study. [file 13089_2019_126_MOESM1_ESM.docx]

**APPENDIX S1**

**Definition of high acuity patients included in this study.**

High acuity patients are defined by using the ABCDE approach to critically ill patients and the following definitions were used at the Emergency Department at Odense University Hospital, Svendborg, Denmark at the time the study was undertaken.

A patient is defined as a high acuity patient if one or more of the following criteria is fulfilled:

**Airways:** Threatened airway with changed voice or inspiratory stridor

**Breathing:** Dyspnoea at rest OR Severe respiratory insufficiency OR peripheral saturation <90% OR RR >30 OR RR< 8

If the patient suffers from chronic obstructive lung disease, the limit for peripheral saturation is <85% without O2 supply or < 90% with O2 supply

**Circulation:** HR >120 OR < 40

OR

Systolic blood pressure < 90mmHg

**Disability:** GCS <14 OR the patient is vigorously agitated or suddenly unclear

OR

The patient has seizures or status epilepticus.

**Exposure:** Tp >40 ^o^C OR < 34^o^C

Abbreviations: O2: Oxygen. RR: Respiratory rate (breaths per minute). HR: Heart rate (beats per minute). GCS: Glasgow Coma Score. Tp: Temperature (^o^C : Degree Celcius).
