# Supplementary material for: Diagnostic value of whole-body-focused ultrasonography in high-acuity patients in the emergency department: a prospective single-center cross-sectional study
Source: Ultrasound J. 2019 May 27;11:11. doi: 10.1186/s13089-019-0126-7 (PMC6638611; doi:10.1186/s13089-019-0126-7)
Supplement: Supplementary file 2 — Additional file 2: Appendix S2. Sonographic definitions and diagnostic criteria of the focused ultrasonography examination. [file 13089_2019_126_MOESM2_ESM.docx]

**APPENDIX S2**

**Sonographic definitions and diagnostic criteria of the focused ultrasonography examination**

These following sonographic definitions and diagnostic criteria are inspired from the sonographic definitions and diagnostic criteria by Laursen et al. [1]. Changes include a reduction in diagnostic measurements in order to make it apply to our study. Thereby the investigators only measure the pericardial effusion but not the ventricle wall or diameter nor the tricuspid annular plane systolic excursion (TAPSE). Small changes have been made in the estimation of the ejection fraction. The diagnostic criteria of the cardiac and pulmonary ultrasonography examination have been reduced and examination of the abdomen has been added.

**Focused Cardiac Ultrasonography (Fcu)**

As previously stated the focused ultrasonography of the lungs is a modification of the ultrasonography protocol used by Laursen et al. [2]. It is originally modified from the principles of lung ultrasonography by Volpicelli and Lichtenstein [3, 4]. The focused echocardiography is performed according to principles described in the international evidence based guideline [5]. Only regular measurements are applied to the presence of pericardial effusion. The remaining criteria are estimated by eyeballing. The following criteria are used as we looked for:

**Pericardial effusion**

Presence of an echo-free zone of > 0,5cm (measured in the diastole) and which separates the pericardium from the heart.

**Left ventricle ejection fraction**

Left ventricle ejection fraction is estimated by “eye-balling” and sub classified into:

Mild left ventricular systolic heart failure: Ejection fraction: 45-65 %

Moderate left ventricular systolic heart failure: Ejection fraction 31-44%

Severe left ventricular systolic heart failure: Ejection fraction ≤ 30%

**Focused Lung Ultrasonography (Flus)**

The definitions and diagnostic criteria for the Flus findings are modified from Laursen et al. [1] who based his modifications on the EFSUMB course book [6]

Modifications consist of: 1) Reduction in subdivisions of ultrasonography findings. 2) A redefinition in the severity of pleural effusion.

To perform a complete Flus we used 7 windows on each hemithorax: 2 anterior, 2 lateral and 3 dorsal.

We looked for:

**Pleural effusion**

The diagnosis of pleural effusion is based on the presence of any echo-free zone separating the visceral and parietal pleura.

**Interstitial syndrome/pulmonary edema**

We use the classification of diffuse interstitial syndrome (IS) which is a pattern that can be seen in a variety of diseases as for example pulmonary oedema, adult respiratory distress syndrome and interstitial lung disease.

Diffuse IS: Presence of multiple (≥3) B-lines in an intercostal space in at least 2 lateral or anterior areas on each side.

**Pneumothorax**

Confirmed pneumothorax: Area with the absence of lung sliding, lung point and B-lines but with the presence of a lung point in an adjacent area.

Suspected pneumothorax: Absence of lung sliding, B-lines, lung pulse and a lung point.

**Focused Abdominal Ultrasonography (Faus)**

The abdominal approach to the patient is based on the assessment of the presence of free intraperitoneal fluid, assessed using the approach of the Focused Assessment by Sonography in Trauma as defined by the RUSH protocol by Perera et al. [7], combined with the assessment of the suspicion or presence of an aorta aneurism or dissection. We used the three abdominal windows: the right upper quadrant, the left upper quadrant and the perivesical window combined with a transverse view of the abdominal aorta sliding the probe from the diaphragm to the bifurcation of the common iliac arteries. Definitions and diagnostic criteria for the abdominal aorta aneurism or dissection are defined in the guidelines from the American College of Emergency Medicine 2006 [8]

We looked for:

**Abdominal aorta aneurism or dissection**

A suspicion of abdominal aorta aneurism or dissection is raised when the diameter of the abdominal aorta measures > 2.9cm in women or > 3.2cm in males.

**Free fluid in the abdomen**

The diagnosis of free intraperitoneal fluid in the abdomen is based on the presence of an echo-free zone located in one of the following areas:

- between the liver and kidney (hepatorenal space or Morison pouch),
- around the spleen (perisplenic space),
- around and behind the bladder (rectovesicular/rectovaginal space or pouch of Douglas

**Limited Compression Ultrasonography (Lcu)**

The method of investigation of a deep vein thrombosis is as made by Perera et al. in the RUSH protocol [7] using a two point compression technique assessing the collapsability of the femoral vein and the popliteal vein.

We looked for:

**Deep vein thrombosis (DVT)**

Suspected DVT: incomplete compression of the anterior and posterior walls of the vein.

Diagnosic criteria of a DVT: An acute blood clot forms a mass in the lumen of the vein, and there is an incomplete compression of the anterior and posterior walls.

**Image Quality**

Upon review by the specialists in ultrasonography the image quality will be graded on a scale from one to five. Each number on the scale is defined as:

1. Poor image quality: it is not possible to recognise any anatomical structures.
2. Impaired image quality: some anatomical structures can be visualised, but it is still not possible to diagnose or exclude any pathology.
3. Suboptimal image quality: some anatomical structures can be visualised, and it is possible to diagnose or exclude rough pathology.
4. Acceptable image quality: all relevant anatomical structures and any potential pathology can be visualised, but still the resolution of the image is not perfect.
5. Excellent image quality: all relevant anatomical structures and any potential pathology can be visualised and the resolution of the picture is near perfect.

**References**

1 Laursen CB, Sloth E, Lassen AT *et al*: Focused sonographic examination of the heart, lungs and deep veins in an unselected population of acute admitted patients with respiratory symptoms: a protocol for a prospective, blinded, randomised controlled trial. *BMJ Open* 2012; 2;(3).

2 Laursen CB, Sloth E, Lassen AT *et al*: Point-of-care ultrasonography in patients admitted with respiratory symptoms: a single-blind, randomised controlled trial. *Lancet Respir Med* 2014; 2;(8):638-46.

3 Volpicelli G, Mussa A, Garofalo G *et al*: Bedside lung ultrasonography in the assessment of alveolar-interstitial syndrome. *Am J Emerg Med* 2006; 24;(6):689-96.

4 Lichtenstein D: General Ultrasonography in the Critically Ill . *Berlin Heidelberg, Germany: Springer-Verlag* 2007.

5 Via G, Hussain A, Wells M *et al*: International evidence-based recommendations for focused cardiac ultrasonography. *J Am Soc Echocardiogr* 2014; 27;(7):683.e1-83.e33.

6 Mathis G: EFSUMB Course Book. 2010;(<http://issuu.com/efsumb/docs/coursebook-chestsono_ch17?e=3336122/6603975> ):Chp 17. UL Chest.

7 Perera P, Mailhot T, Riley D *et al*: The RUSH exam: Rapid Ultrasonography in SHock in the evaluation of the critically lll. *Emerg Med Clin North Am* 2010; 28;(1):29-56, vii.

8 Emergency ultrasonography imaging criteria compendium. American College of Emergency Physicians. *Ann Emerg Med* 2006; 48;(4):487-510.
