# Supplementary material for: Diagnostic value of whole-body-focused ultrasonography in high-acuity patients in the emergency department: a prospective single-center cross-sectional study
Source: Ultrasound J. 2019 May 27;11:11. doi: 10.1186/s13089-019-0126-7 (PMC6638611; doi:10.1186/s13089-019-0126-7)
Supplement: Supplementary file 4 — Additional file 4. Number of pathological ultrasonography findings compared to gold standard diagnoses in the adult high acuity patients admitted to the emergency department. [file 13089_2019_126_MOESM4_ESM.docx]

| **Additional file 4** |  |  |  |  | |  | |  | |  | |  | |  |
| --- | --- | --- | --- | --- | --- | --- | --- | --- | --- | --- | --- | --- | --- | --- |
|  |  |  |  |  | |  | |  | |  | |  | |  |
| Number of pathological ultrasonography findings compared to gold standard diagnoses in the adult high acuity patients admitted to the emergency department. | | | | | | | | | | | | | | |
|  |  |  |  |  |  | |  | |  | |  | |  | |
|  |  |  |  |  |  | |  | |  | |  | |  | |
| **Gold standard diagnoses** | **Prevalence of gold standard** | **F-us: pericardial effusion** | **F-us: systolic heart failure EF< 45%** | **F-us: pneumothorax** | **F-us: pleural effusion** | | **F-us: interstitiel lung syndrome/pulmonary edema** | | **F-us: signs of abdominal aorta aneurism/dissection** | | **F-us: free fluid abdomen** | | **F-us: signs of DVT** | |
|  | **N=171** | **N=144** | **N=144** | **N=155** | **N=157** | | **N=158** | | **N= 130** | | **N=145** | | **N=128** | |
| **Cerebral diseases** |  |  |  |  |  | |  | |  | |  | |  | |
| Cerebral stroke | 12 | 1 | 1 |  | 1 | | 1 | |  | |  | |  | |
| Cerebral hemorrhagia | 2 |  |  |  |  | | 1 | |  | |  | |  | |
| **Pulmonary diseases** |  |  |  |  |  | |  | |  | |  | |  | |
| COPD | 2 |  |  |  |  | |  | |  | |  | |  | |
| COPD with suspected exacerbation | 5 |  |  |  | 7 | | 4 | | 1 | |  | |  | |
| Suspected COPD with exacerbation | 15 |  |  |  | 1 | |  | |  | |  | |  | |
| Asthma with exacerbation | 4 |  |  |  |  | |  | |  | | 1 | |  | |
| Asthma without exacerbation | 0 |  |  |  |  | |  | |  | |  | |  | |
| Interstital lung disease | 3 |  |  |  |  | | 3 | |  | |  | |  | |
| Pneumonia | 24 | 5 | 6 |  | 13 | | 11 | |  | | 1 | |  | |
| Pneumothorx* | 0 |  |  |  |  | |  | |  | |  | |  | |
| Pleural effusion* | 22 | 2 | 6 |  | 17 | | 9 | |  | | 1 | |  | |
| Pulmonary edema* | 6 |  | 1 |  | 6 | | 3 | |  | | 1 | |  | |
| Parapneumonic effusion | 6 |  | 3 |  | 6 | | 3 | |  | | 1 | |  | |
| Empyema | 0 |  |  |  |  | |  | |  | |  | |  | |
| Pulmonary embolism | 3 |  |  |  | 1 | |  | |  | |  | |  | |
| **Heart diseases** |  |  |  |  |  | |  | |  | |  | |  | |
| Systolic heart failure* | 4 |  | 2 |  | 4 | | 2 | |  | | 1 | |  | |
| Nonsystolic heart failure | 2 |  |  |  | 2 | | 1 | |  | |  | |  | |
| Myocardial infarction (acute or recently evolved) | 2 | 1 | 2 |  | 1 | | 1 | |  | |  | |  | |
| MI established | 0 |  |  |  |  | |  | |  | |  | |  | |
| Pericardial infusion* | 2 | 2 |  |  | 2 | |  | |  | |  | |  | |
| Valvular heart disease | 12 | 2 | 3 |  | 7 | | 4 | |  | |  | |  | |
| Infective endocarditis | 0 |  |  |  |  | |  | |  | |  | |  | |
| Cardiac arrythmia | 26 | 2 | 7 |  | 11 | | 8 | |  | | 1 | |  | |
| Chest pain (myocardial infarction ruled out | 9 |  | 2 |  | 1 | |  | |  | |  | |  | |
|  |  |  |  |  |  | |  | |  | |  | |  | |
| **Abdominal diseases** |  |  |  |  |  | |  | |  | |  | |  | |
| Ileus | 0 |  |  |  |  | |  | |  | |  | |  | |
| Appendicitis | 2 |  |  |  |  | |  | |  | |  | |  | |
| Dissection/aneurism of the abdominal aorta* | 0 |  |  |  |  | |  | |  | |  | |  | |
| Pancreatitis/kidney stone | 8 | 2 |  |  | 1 | | 1 | |  | |  | |  | |
| Free fluid abdomen* | 1 |  |  |  |  | |  | |  | | 1 | |  | |
| **Orthopedic diaseses** |  |  |  |  |  | |  | |  | |  | |  | |
| Fracture | 8 |  | 3 |  | 3 | |  | |  | |  | |  | |
| Luxation | 5 |  | 1 |  |  | |  | |  | |  | |  | |
| Orthopedic lesions (no fracture/luxation) | 6 |  |  |  |  | |  | |  | |  | |  | |
| **Other diseases or symptoms** |  |  |  |  |  | |  | |  | |  | |  | |
| Infection with extra pulmonary focus | 31 | 3 | 4 |  | 6 | | 5 | |  | | 2 | |  | |
| DVT* | 0 |  | 2 |  | 4 | | 5 | |  | | 1 | |  | |
| Anemia | 14 |  |  |  |  | |  | |  | |  | |  | |
| Malignancy | 7 | 1 |  |  |  | | 2 | |  | |  | |  | |
| lipotymi/dizziness/discomfort | 10 |  |  |  | 1 | |  | |  | |  | |  | |
| Poisoning | 4 |  |  |  | 1 | |  | |  | |  | |  | |
| **No diagnostic criteria met** | 28 | 1 |  |  | 2 | |  | |  | | 1 | |  | |
|  |  |  |  |  |  | |  | |  | |  | |  | |
|  |  |  |  |  |  | |  | |  | |  | |  | |
|  |  |  |  |  |  | |  | |  | |  | |  | |
|  |  |  |  |  |  | |  | |  | |  | |  | |
|  |  |  |  |  |  | |  | |  | |  | |  | |
|  |  |  |  |  |  | |  | |  | |  | |  | |
|  |  |  |  |  |  | |  | |  | |  | |  | |

**Abbreviations:** COPD: Chronic obstructive pulmonary disease. DVT: Deep vein thrombosis**,** F-us: focused ultrasonography. N= number of patients investigated.
