# Supplementary material for: Diagnostic value of whole-body-focused ultrasonography in high-acuity patients in the emergency department: a prospective single-center cross-sectional study
Source: Ultrasound J. 2019 May 27;11:11. doi: 10.1186/s13089-019-0126-7 (PMC6638611; doi:10.1186/s13089-019-0126-7)
Supplement: Supplementary file 5 — Additional file 5. a Per protocol correlation and diagnostic accuracy between investigators pathological ultrasonography findings and gold standard pathology found in adult high acuity patients admitted to the emergency department. Contingency tables and calculations are made according to the statistical concept ‘per protocol’, hence only including the patients who received a focused ultrasonography examination. b Per intention to treat correlation and diagnostic accuracy between investigators pathological ultrasonography and gold standard pathology found in adult high acuity patients admitted to the emergency department. Contingency tables and calculations are made according to the statistical concept `intention to treat´ including the entire study population. N = 171. [file 13089_2019_126_MOESM5_ESM.docx]

**Additional file 5a**

**Per protocol**

Correlation and diagnostic accuracy between investigators pathological ultrasonography findings and gold standard pathology found in adult high acuity patients admitted to the emergency department. Contingency tables and calculations are made according to the statistical concept `per protocol´, hence only including the patients who received a focused ultrasonography examination.

**Pericardial effusion**

| **Investigator** | **Gold standard** | | **Total** |
| --- | --- | --- | --- |
|  | **1** | **0** |  |
| **1** | 2 | 8 | 10 |
| **0** | 0 | 134 | 134 |
| **Total** | 2 | 142 | 144 |

Sensitivity 100 % (CI 16-100%), specificity 94% (CI 89-98%),

PPV 20% (CI 3-56%), NPV 100% (CI 97-100%).

**Systolic heart failure**

| **Investigator** | **Gold standard** | | **Total** |
| --- | --- | --- | --- |
|  | **1** | **0** |  |
| **1** | 2 | 17 | 19 |
| **0** | 2 | 123 | 125 |
| **Total** | 4 | 140 | 144 |

Sensitivity 50% (CI 7-93 %), specificity 88% (CI 81-93%),

PPV 11 % (CI 1-33 %), NPV 98% (CI 94-100%).

**Pneumothorax**

| **Investigator** | **Gold standard** | | **Total** |
| --- | --- | --- | --- |
|  | **1** | **0** |  |
| **1** | 0 | 0 | 0 |
| **0** | 0 | 155 | 155 |
| **Total** | 0 | 155 | 155 |

*

**Pleural effusion**

| **Investigator** | **Gold standard** | | **Total** |
| --- | --- | --- | --- |
|  | **1** | **0** |  |
| **1** | 17 | 22 | 39 |
| **0** | 2 | 116 | 118 |
| **Total** | 19 | 138 | 157 |

Sensitivity 89% (CI 67-99%), specificity 84% (CI 77-90%),

PPV 44% (CI 28-60%), NPV 98% (CI 94-100%).

**Pulmonary edema**

| **Investigator** | **Gold standard** | | **Total** |
| --- | --- | --- | --- |
|  | **1** | **0** |  |
| **1** | 3 | 22 | 25 |
| **0** | 3 | 130 | 133 |
| **Total** | 6 | 152 | 158 |

Sensitivity 50% (CI 12-88%), specificity 86% (CI 79-91%),

PPV 12% (CI 3-31%), NPV 98% (CI 94-100%).

**Interstitial lung disease**

| **Investigator** | **Gold standard** | | **Total** |
| --- | --- | --- | --- |
|  | **1** | **0** |  |
| **1** | 3 | 22 | 25 |
| **0** | 0 | 133 | 133 |
| **Total** | 3 | 155 | 158 |

Sensitivity 100% (CI 29-100%), specificity 86% (CI 79-91%),

PPV 12% (CI 3-31%), NPV 100% (CI 97-100%).

**Abdominal aorta aneurism or dissection**

| **Investigator** | **Gold standard** | | **Total** |
| --- | --- | --- | --- |
|  | **1** | **0** |  |
| **1** | 0 | 1 | 1 |
| **0** | 0 | 129 | 129 |
| **Total** | 0 | 130 | 130 |

Sensitivity 100% (CI 3-100%), specificity 98% (CI 94-100%),

PPV 25% (CI 1-81%), NPV 100% (CI 97-100%).

**Free fluid abdomen**

| **Investigator** | **Gold standard** | | **Total** |
| --- | --- | --- | --- |
|  | **1** | **0** |  |
| **1** | 1 | 3 | 4 |
| **0** | 0 | 141 | 141 |
| **Total** | 1 | 144 | 145 |

Sensitivity 100% (CI 3-100%), specificity 98% (CI 94-100%)

PPV 25% (CI 1-81%), NPV 100% (CI 97-100%)

**Deep vein thrombosis**

| **Investigator** | **Gold standard** | | **Total** |
| --- | --- | --- | --- |
|  | **1** | **0** |  |
| **1** | 0 | 0 | 0 |
| **0** | 0 | 128 | 128 |
| **Total** | 0 | 128 | 128 |

*****

**Abbreviations:** PPV (positive predictive value), NPV (negative predictive value), CI (95% confidence interval).

*Due to a low number of events diagnostic accuracy values could not be calculated.

**Additional file 5b**

**Intention to treat**

Correlation and diagnostic accuracy between investigators pathological ultrasonography findings and gold standard pathology found in adult high acuity patients admitted to the emergency department. Contingency tables and calculations are made according to the statistical concept `intention to treat´ including the entire study population. N = 171.

**Pericardial effusion**

| **Investigator** | **Gold standard** | | **Total** |
| --- | --- | --- | --- |
|  | **1** | **0** |  |
| **1** | 2 | 8 | 10 |
| **0** | 0 | 161 | 161 |
| **Total** | 2 | 169 | 171 |

Sensitivity 100 % (CI 16-100%), specificity 94% (CI 84-98%),

PPV 20% (CI 3-56%), NPV 100% (CI 97-100%).

**Systolic heart failure**

| **Investigator** | **Gold standard** | | **Total** |
| --- | --- | --- | --- |
|  | **1** | **0** |  |
| **1** | 2 | 17 | 19 |
| **0** | 2 | 150 | 152 |
| **Total** | 4 | 167 | 171 |

Sensitivity 50% (CI 7-93 %), specificity 88% (CI 81-93%),

PPV 11 % (CI 1-33 %), NPV 98% (CI 94-100%).

**Pneumothorax**

| **Investigator** | **Gold standard** | | **Total** |
| --- | --- | --- | --- |
|  | **1** | **0** |  |
| **1** | 0 | 0 | 0 |
| **0** | 0 | 171 | 171 |
| **Total** | 0 | 171 | 171 |

*

**Pleural effusion**

| **Investigator** | **Gold standard** | | **Total** |
| --- | --- | --- | --- |
|  | **1** | **0** |  |
| **1** | 17 | 22 | 39 |
| **0** | 5 | 127 | 132 |
| **Total** | 22 | 149 | 171 |

Sensitivity 90% (CI 67-99%), specificity 94% (CI 89-97%),

PPV 44% (CI 28-60%), NPV 98% (CI 94-100%).

**Pulmonary edema**

| **Investigator** | **Gold standard** | | **Total** |
| --- | --- | --- | --- |
|  | **1** | **0** |  |
| **1** | 3 | 22 | 25 |
| **0** | 3 | 143 | 146 |
| **Total** | 6 | 165 | 171 |

Sensitivity 50% (CI 12-88%), specificity 85% (CI 78-90%),

PPV 12% (CI 3-31%), NPV 98% (CI 93-100%).

**Interstitial lung disease**

| **Investigator** | **Gold standard** | | **Total** |
| --- | --- | --- | --- |
|  | **1** | **0** |  |
| **1** | 3 | 22 | 25 |
| **0** | 0 | 146 | 146 |
| **Total** | 3 | 168 | 171 |

Sensitivity 100% (CI 29-100%), specificity 85% (CI 79-91%),

PPV 12% (CI 13-31%), NPV 100% (CI 97-100%).

**Abdominal aorta aneurism or dissection**

| **Investigator** | **Gold standard** | | **Total** |
| --- | --- | --- | --- |
|  | **1** | **0** |  |
| **1** | 0 | 1 | 1 |
| **0** | 0 | 170 | 170 |
| **Total** | 0 | 171 | 171 |

*****

**Free fluid abdomen**

| **Investigator** | **Gold standard** | | **Total** |
| --- | --- | --- | --- |
|  | **1** | **0** |  |
| **1** | 1 | 3 | 4 |
| **0** | 0 | 167 | 167 |
| **Total** | 1 | 170 | 171 |

Sensitivity 100% (CI 3-100%), specificity 98% (CI 95-100%),

PPV 25% (CI 1-81%), NPV 100% (CI 98-100%).

**Deep vein thrombosis**

| **Investigator** | **Gold standard** | | **Total** |
| --- | --- | --- | --- |
|  | **1** | **0** |  |
| **1** | 0 | 0 | 0 |
| **0** | 0 | 171 | 171 |
| **Total** | 0 | 171 | 171 |

*****

**Abbreviations:** PPV (positive predictive value), NPV (negative predictive value), CI (95% confidence interval).

*Due to a low number of events diagnostic accuracy values could not be calculated.
